# Manipulating the physical distance between cells during soil colonization reveals the importance of biotic interactions in microbial community assembly

**DOI:** 10.1186/s40793-024-00559-4

**Published:** 2024-03-19

**Authors:** Sana Romdhane, Sarah Huet, Aymé Spor, David Bru, Marie-Christine Breuil, Laurent Philippot

**Affiliations:** Univ. Bourgogne Franche-Comté, INRAE, Institut Agro, Agroécologie, F-21000 Dijon, France

**Keywords:** Microbial interactions, Community manipulation, Coalescence, Microbial assembly

## Abstract

**Background:**

Microbial communities are of tremendous importance for ecosystem functioning and yet we know little about the ecological processes driving the assembly of these communities in the environment. Here, we used an unprecedented experimental approach based on the manipulation of physical distance between neighboring cells during soil colonization to determine the role of bacterial interactions in soil community assembly. We hypothesized that experimentally manipulating the physical distance between bacterial cells will modify the interaction strengths leading to differences in microbial community composition, with increasing distance between neighbors favoring poor competitors.

**Results:**

We found significant differences in both bacterial community diversity, composition and co-occurrence networks after soil colonization that were related to physical distancing. We show that reducing distances between cells resulted in a loss of bacterial diversity, with at least 41% of the dominant OTUs being significantly affected by physical distancing. Our results suggest that physical distancing may differentially modulate competitiveness between neighboring species depending on the taxa present in the community. The mixing of communities that assembled at high and low cell densities did not reveal any “home field advantage” during coalescence. This confirms that the observed differences in competitiveness were due to biotic rather than abiotic filtering.

**Conclusions:**

Our study demonstrates that the competitiveness of bacteria strongly depends on cell density and community membership, therefore highlighting the fundamental role of microbial interactions in the assembly of soil communities.

**Supplementary Information:**

The online version contains supplementary material available at 10.1186/s40793-024-00559-4.

## Background

Understanding community assembly processes is one of the fundamental goals in community ecology. Although soil microbial communities play an essential role in several key ecosystem functions such as biogeochemical cycling, plant productivity and carbon sequestration [1–3], it is unclear what process, or combinations of processes, are driving their composition. For example, much focus has been laid on the role of environmental filters in shaping microbial communities [4] while how interactions among microorganisms drive their assembly remains largely unexplored [5]. However, a growing body of evidence suggests that these biotic interactions may also play an important role in microbial community assembly [6–8].

Most microorganisms face a constant battle for resources and a large range of interactions between microorganisms has been reported [9]. For example, competitive interactions can occur through either exploitative or interference mechanisms [10]. Exploitative competition is an indirect mechanism in which the consumption of limiting resources by one strain restricts its supply to competitors, while interference competition is a direct mechanism involving the production of antimicrobial compounds (e.g. antibiotics, toxins) to harm competitors [11]. On the opposite, positive interactions include tightly coupled mutualistic interactions such as syntrophy, in which both partners depend on each other to perform a metabolic activity [12]. Whether positive or negative, interactions mostly occur between individuals that are close in space. In some cases, a physical contact between cells is even required for the injection of secreted toxins to the rival strain as exemplified by the type VI secretion system, which mediates interactions between a broad range of Gram-negative bacteria. In soil, it has been estimated that a single bacterium has about 120 neighboring species within interaction distance [13]. Yet, we still lack a clear understanding of how prevalent these interactions are, and how they affect community composition.

Theory predicts that in a new environment and without immediate neighbors, microorganisms will first colonize the empty space, which can be considered as a surrogate limiting resource [14, 15]. After this initial phase of range expansion, direct interactions at the boundaries between neighboring patches of different species will emerge and affect the speed of expansion [16, 17]. In ecology, the competition-colonization trade-off is a fundamental mechanism proposed to explain species coexistence, where better competitors are inferior colonizers and vice versa [18, 19]. It can therefore be expected that the physical distance between species during the range expansion phase is of importance, as shorter distances will favor species with superior competitive abilities over species with superior colonization abilities [20, 21]. Although models have been used to explore how the density of surrounding neighbors influences biotic interactions [22–24], we are not aware of any study that has explicitly tested how changing physical distance between microbes in a complex environment affect the outcome of competition for space.

**Fig. 1:**
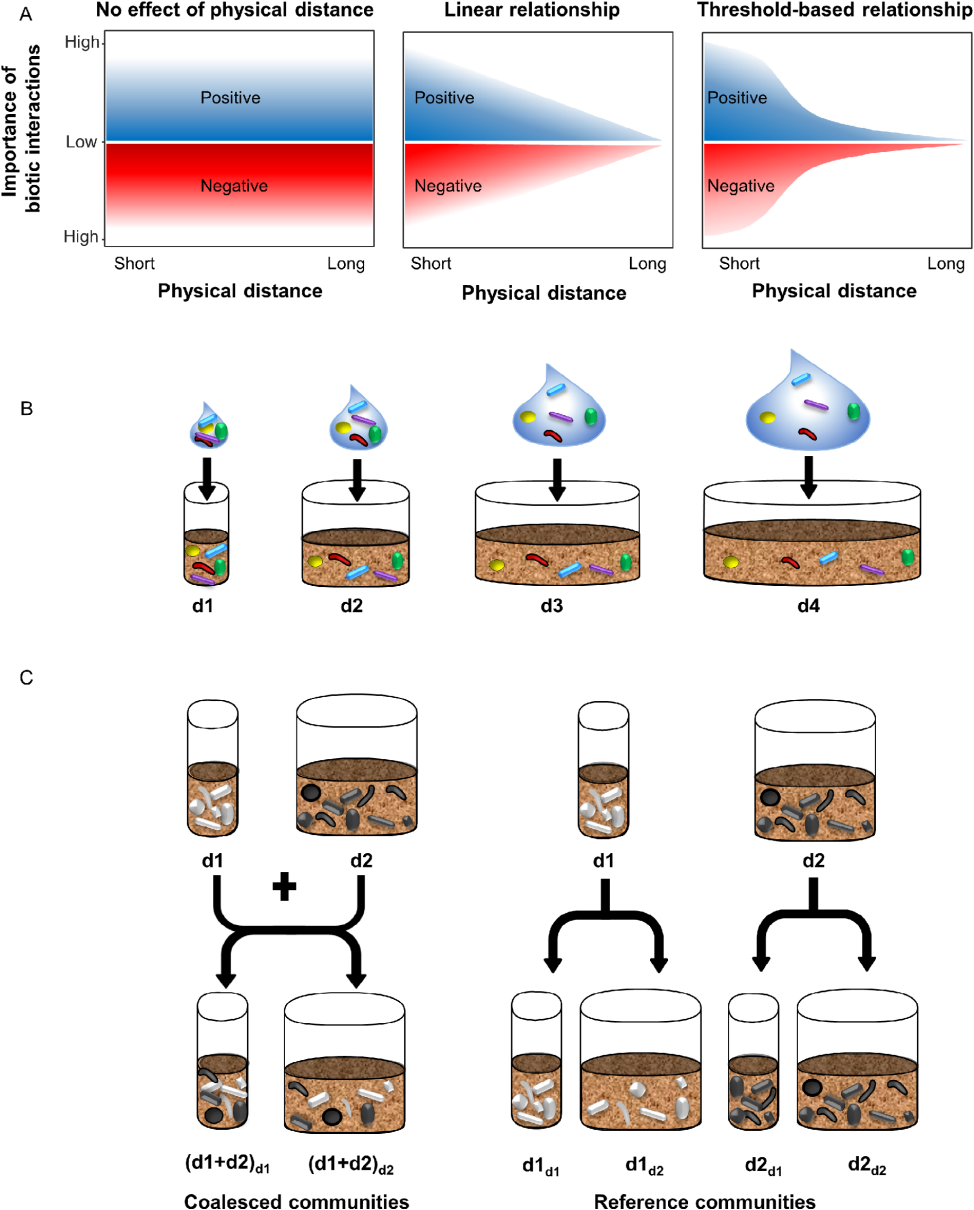
Schematic illustration of the experimental design. (A) Examples of possible scenarios outlining the impact of increasing physical distance between the neighboring cells (x-axis) on the importance of biotic interactions between microbial species (y-axis). Negative and positive interactions are represented in red and blue, respectively. (B) In the first step, the physical distance between microbial cells was experimentally manipulated by introducing the same species pool into increasing volumes of sterilized soil (*n* = 10) for control, heat-shock and ramoplanin communities. (C) Step 2 consisted in a coalescence experiment with a reciprocal transplant design by mixing microbial communities that assembled at high (d1) or low (d2) densities during step1 in sterile soil (coalesced communities). Soils colonized under the d1 and the d2 initial densities were also incubated separately with sterile soil at high (d1) or low (d2) densities (reference communities)

Here we examine to which extent the distance between neighboring species in soil determine their competitiveness for a more general understanding of the role of biotic interactions in microbial community assembly. We hypothesized that experimentally manipulating the physical distance between microbial cells will modify the interaction frequency leading to differences in microbial community composition. For example, an increase in the initial physical distance between neighboring cells would reduce the interaction frequency, and thus favor poor competitors in the community (Fig. 1A). Since both the type and the importance of interactions vary between taxa [25–27], we also hypothesized that the importance of physical distance in shaping microbial communities will be modulated by community membership (i.e. the taxa present in the community). To generate microbial community inoculums with noticeable variation in membership, we first subjected a soil microbial community to two removal treatments, exposure to heat-shock or ramoplanin, which are depleting Gram-negative and Gram-positive bacteria, respectively [8]. These treatments were selected because of the distinctive traits between Gram-negative and Gram-positive taxa that can affect their competitiveness. For example, Gram-negative bacteria possess an additional outer membrane, which increases resistance to antibiotics [28]. The contact-dependent antagonistic type VI secretion system is also only present in Gram-negative bacteria [29]. In addition, Gram-negative bacteria rely on acylated homoserine lactones as cell density-dependent quorum-sensing signaling molecules, while Gram-positive bacteria are mostly using oligopeptides [30, 31]. We then experimentally manipulated the physical distance between microbial cells by inoculating the same species pool (control, heat-shock and ramoplanin treated microbial communities), into increasing volumes of sterilized soil (Fig. 1B). Finally, we performed a coalescence experiment with a reciprocal transplant design by mixing microbial communities that assembled with different initial distances between cells in order to assess their relative competitiveness (Fig. 1C).

## Methods

### Soil sampling and experimental design

The soil was collected from the Epoisses experimental farm in France (47° 30′ 22.1832′′ N, 4° 10′ 26.4648′′ E) in October 2019 and sieved through 4 mm. The soil properties were 41.9% clay, 51.9% silt, and 6.2% sand, pH 7.2 (pH in water measured according to the ISO 10,390 standard), C and N content 15.5 and 1.4 g.kg^− 1^ dry soil, respectively. The soil was γ-sterilized (70 kGy at Conservatome, Dagneux, France) and used to prepare microcosms with 4 different soil volumes (i.e. d1 = 6 g, d2 = 43 g, d3 = 134 g and d4 = 508 g) corresponding to distinct diameters (i.e. d1 = 2 cm, d2 = 5.4 cm, d3 = 9.5 cm and d4 = 18.5 cm) in order to obtain the same soil depth (2 cm) in all microcosms. Each microcosm volume was replicated 10 times. Soil suspensions were prepared by initially adding 100 g equivalent dry mass of fresh soil to 150 ml sterile distilled water. This mixture was blended using a Waring blender and then subjected to a tenfold dilution under sterilized conditions. Variation in community memberships was induced through the heat-shock (HS) and the biocidal antibiotic ramoplanin (RA) removal treatments, which are depleting Gram-negative and Gram-positive bacteria, respectively. The HS treatment was applied as follows: 0 °C for 5 min / 70 °C for 15 min / 0 °C for 5 min, and the RA treatment was applied at a concentration of 70 µg mL^− 1^ of soil suspension. Non-treated soil suspensions were used as controls (C). To manipulate physical distance between neighboring cells, we diluted the same volume of soil suspensions (i.e. 1 mL of HS, RA or C) into 4 different volumes of water calculated in order to reach the same soil moisture of 30% after inoculating the d1, d2, d3 and d4 sterilized soil microcosms (i.e. V1 = 1.25 mL, V2 = 9.14 mL, V3 = 28.21 mL, and V4 = 106.89 mL). Then, the entire volume of each diluted soil suspensions were inoculated into the different microcosms so that inoculated microcosms contained the same number of cells and the same species pool, with the same soil moisture (Fig. 1B). The soil suspensions were thoroughly vortexed and equally distributed on the entire soil surface whatever the microcosms diameter, therefore resulting in a gradient of cell density per gram soil. In the control treatment, the estimated initial densities calculated as 16S rRNA gene copies g^− 1^ dry soil were: d1 = 4.3 × 10^6^, d2 = 6 × 10^5^, d3 = 1.9 × 10^5^ and d4 = 5.1 × 10^4^. The microcosms were then sealed with Parafilm allowing gas exchange in aseptic conditions and incubated at 20 °C for 4 months. After incubation, 130 soil samples, comprising soil microcosms with the C, HS, and RA communities at 4 different initial distances (*n* = 10 for a total of 120 samples) and the original soils (*n* = 10) were used for subsequent analyses. In a second step, we selected the control (C) and heat-shock (HS) communities from d1 and d2 microcosms (i.e. Cd1, Cd2, HSd1 and HSd2) for the coalescence experiment as these communities were the most dissimilar after the first step. For this purpose, 1.5 g of soil from microcosm colonized under the d1 density was thoroughly mixed with 1.5 g of soil from microcosm colonized under the d2 density into either 3 or 43 g of sterile soil microcosm to again obtain microcosms with short (d1) or long (d2) physical distancing between cells (Fig. 1C). Soils from Step 1 selected communities (i.e. Cd1, Cd2, HSd1 and HSd2) were also mixed only with sterile soil at the d1 and d2 densities to obtain reference communities. Soil microcosms from Step 2 were replicated 5 times and incubated under the same condition as Step 1 for 90 days (a total of 60 soil microcosms).

### Assessment of microbial community composition and diversity

Before DNA extraction, the entire soil from each microcosm was manually homogenized by thorough mixing. DNA was extracted from 190 samples (ten original soil samples, 120 Step 1 microcosms and 60 Step 2 microcosms) using the DNeasy PowerSoil-htp 96 well DNA isolation kit (Qiagen, France) according to the manufacturer’s instructions. To generate amplicons, a 2-step PCR approach was used according to [32]. The V3-V4 hypervariable region of the 16S rRNA gene was amplified using the 341F (5’-CCTACGGGRSGCAGCAG-3’) and 805R (5’-GACTACCAGGGTATCTAAT-3’) [33]. The amplicon size was checked with 2% agarose gel and DNA concentration was estimated using Quant-IT™ dsDNA HS Assay kit (Invitrogen™, Carlsbad, CA, USA). Final PCR products were purified and their concentration normalized using the SequalPrep Normalization plate kit (Invitrogen™, Carlsbad, CA, USA). Sequencing was performed on MiSeq (Illumina, 2 × 250 bp amplicons) using the MiSeq reagent kit v2. Demultiplexing and trimming of Illumina adaptors and barcodes was done with Illumina MiSeq Reporter software (version 2.5.1.3). Sequence data from soil samples were analysed using an in-house developed Python pipeline (available upon request). Briefly, 16S rRNA gene sequences were assembled using PEAR [34] with default settings. Further quality checks were conducted using the QIIME pipeline [35] and short sequences were removed (< 400 bp). Reference based and *de novo* chimera detection, as well as OTUs clustering were performed using VSEARCH [36] and the adequate reference databases (SILVA’ representative set of sequences from Quast et al., 2013). The identity thresholds were set at 94% based on replicate sequencing of a bacterial mock community [8]. Representative sequences for each OTU were aligned using Infernal [37] and phylogenetic trees were construct using FastTree [38]. Taxonomy was assigned using UCLUST [39] and the SILVA database (138.1/2020) [40]. Raw sequences were deposited at the NCBI under the BioProject PRJNA883551.

### Quantification of microbial communities

The abundances of the total bacterial community were estimated by real-time quantitative PCR (qPCR) assays. For each treatment, we used five equimolar mixtures prepared from pairs of the 10 DNA replicates extracts. The total bacterial community was quantified using 16S rRNA primers as described by Muyzer et al. [33]. Real-time qPCR assays were carried out in a ViiA7 (Life Technologies, USA) with a Takyon Master Mix (Eurogentec, France) as previously described [41]. An average PCR efficiency of 100.7% was found for the two independent runs. No-template controls gave null or negligible values. PCR inhibitor presence was tested by mixing soil DNA extracts with either control plasmid DNA (pGEM-T Easy Vector, Promega, France) or water. No inhibition was detected in any case.

### Statistical analysis

Statistical analyses were conducted using R statistical software version 4.0.3 [42]. Bacterial α-diversity metrics (i.e. observed species, Simpson’s reciprocal, Shannon and Faith’s Phylogenetic Diversity PD from [43]) and Weighted Unifrac distance [44] between samples were calculated based on rarefied OTU Table (12,000 sequences). Differences between treatments in gene copy abundances (16S rRNA) (*n* = 5) and the microbial α-diversity indices (*n* = 10) were tested using ANOVAs followed by Tukey’s honestly significant difference (HSD) test (p-value ≤ 0.05) using the agricolae package version 1.3-5 [45]. Normality and homogeneity of the residual distribution were verified, and log-transformations were performed for gene copy abundances. Differences between Weighted Unifrac distances were tested using a Kruskal-Wallis test followed by a Nemenyi’s all-pairs comparison test (p-value ≤ 0.05) using the PMCMRplus package (version 1.9.4). We also performed principal coordinates analysis (PCoA) based on the Weighted Unifrac distance matrix to detect changes in the microbial community structure and a Permutational multivariate analysis of variance (PERMANOVA) from [46] to detect significant differences between treatments using the adonis function of the vegan package (version 2.5-7). Pairwise post hoc tests were conducted using the function pairwise.adonis from the pairwiseAdonis package with Benjamini–Hochberg corrections [47].

#### Identification of differentially abundant OTUs in treatments

Low-abundance OTUs were filtered out by keeping OTUs that (i) represented > 0.01% of the sequences across all samples and (ii) were found in at least 60% of the replicates, which resulted in 792 OTUs. Due to differences in community composition between control and removal treatments, OTUs with low prevalence (i.e. present in less than 50% of replicates within each removal treatment or control) were removed which resulted in the Step 1 experiment in 529, 306 and 468 OTUs for C, HS and RA communities, respectively, and in the Step 2 experiment in 495 and 323 OTUs for C and HS communities, respectively. To estimate differences in OTUs abundances between treatments, we used a generalized linear mixed model (GLMM). Such model combines a generalized linear model, which allow to infer linear regression from data that does not follow a normal distribution as abundance data typically follow a Poisson distribution, with a mixed model which contain both fixed effects (treatment effects) and random effects (sampling effects). We considered that an OTU of abundance $$ Y$$ follows a Poisson law of parameter $$ {\Lambda }$$ as $$ \text{Y}\sim \mathcal{P}\left(\varLambda \right)$$, in any $$ j$$ replicates of any $$ i$$ treatment. Thus, we used the following model (Eq. 1):

1 $$ \text{log}\left({{\Lambda }}_{ij}\right)={o}_{ij}+{\upmu }+{\alpha }_{i}+{Z}_{ij}, {{Z}_{ij}}_{1\le j\le 5}\text{ iid}\sim \mathcal{N}\left(0,{\sigma }^{2}\right)$$

where $$ o$$ is the offset for each sample calculated as the log of the sample read sum, $$ \alpha $$ is the effect of the treatments, and$$ Z$$ is the random sampling effect modeling the data overdispersion. For the Step 1 experiment, $$ i=\left\{1,\dots,4\right\}$$ represents the density treatments of either one removal treatment or control, and $$ j=\{1,\dots,10\}$$ represents the replicates. For the Step 2 experiment, $$ i=\left\{1,\dots,6\right\}$$ represents the coalescence and self-mixed treatments of either removal treatment or control, and $$ j=\{1,\dots,5\}$$ represents the replicates. The analysis was performed using the glmer function of the *lme4* package (version 1.1–27). Subsequently, we performed a post-hoc Tukey test with the emmeans function of the *emmeans* package (version 1.6.1). Thereby, we implemented multiple pairwise comparisons for each OTU (i) between density treatments within each Step 1 removal treatment or control and (ii) between each coalesced community and its references communities within each Step 2 removal treatment or control. The p-values were then adjusted using the false discovery rate (FDR) method [48]. Only OTUs with FDR adjusted p-values below or equal to 0.05 and with coefficient estimates higher or equal to 0.5 were considered significant.

#### Inference of co-occurrence networks

Networks were constructed based on the most abundant OTU count data (low-abundance OTUs filtered out) from the Step 1 experiment, and individually built using samples from each C, HS and RA communities (*n* = 40). Networks were inferred using a sparse multivariate Poisson log-normal (PLN) model with a latent Gaussian layer and an observed Poisson layer using the PLNmodels package, which allows to account for offsets and covariates [49]. A specific normalization corresponding to the log-transformed number of reads in each sample was added as an offset in order to take into account the heterogeneity of sequencing depth. For each sample set, we constructed networks using two distinct models: a null model without the physical distance as a covariate (M0), and a full model that included physical distance as a covariate (M1). The integration of the physical distance as a covariate in the model M1 allows the identification of links/nodes which are not related to this covariate. This comparative analysis using a dual-model approach aimed to identify nodes and links specifically associated to the effect of the physical distancing treatment. For each model, the best network was selected using a Stability Approach to Regularization Selection (StARS) [50], which performs a random subsampling of the input data to evaluate the robustness of the network selected edges.

## Results

### Manipulating cell density alters the diversity and composition of the bacterial community

**Fig. 2:**
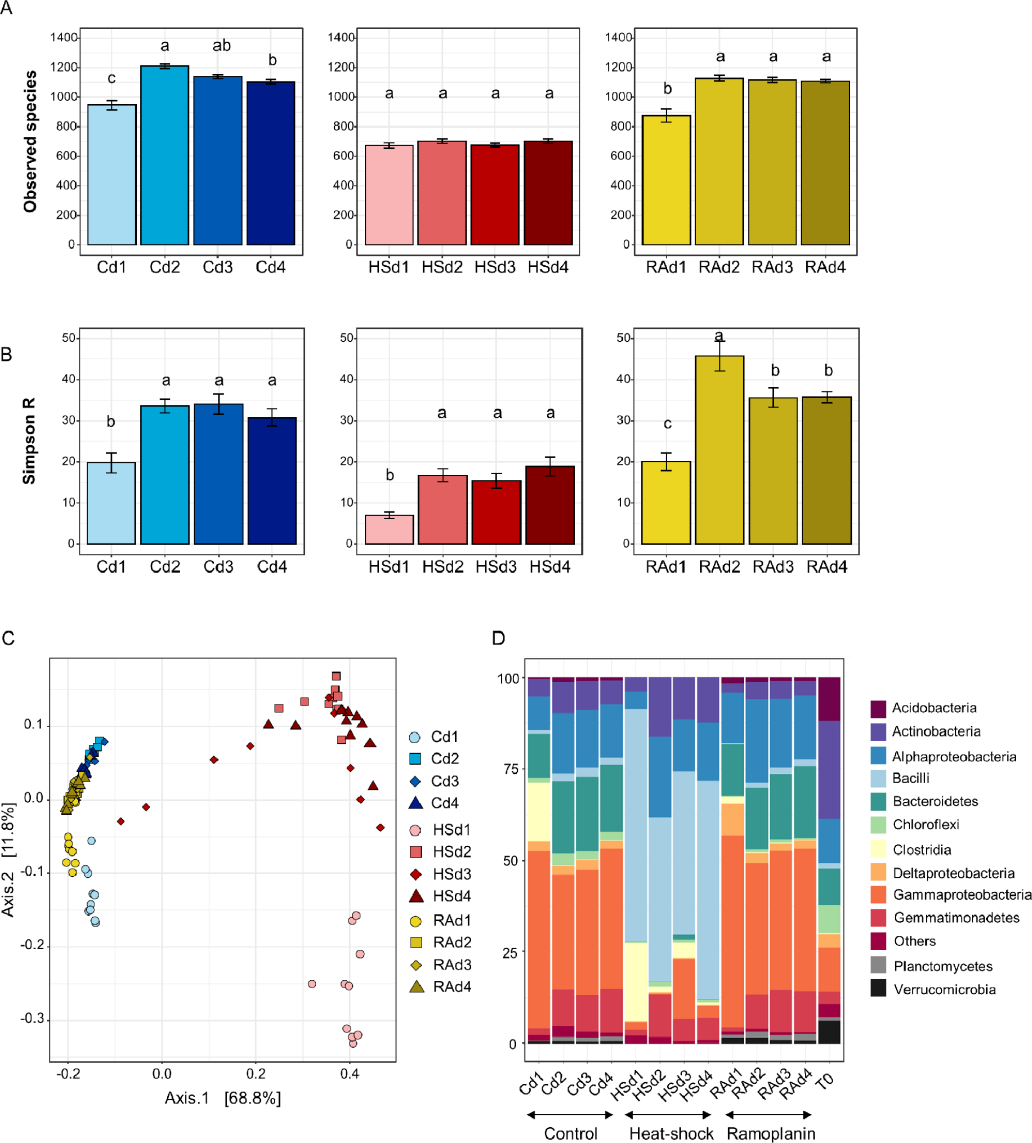
Differences in bacterial community diversity, structure and composition across density treatments after the step 1 experiment. (A) Observed species and (B) Simpson’s reciprocal indices are shown (mean ± s.e.) in the control (C), heat-shock (HS) and ramoplanin (RA) communities within the density gradient (d1, d2, d3 and d4). Different letters indicate significant differences according to TukeyHSD test (p-value < 0.05). (C) Principal Coordinates Analysis (PCoA) of the weighted UniFrac distance matrix of 16S rRNA gene amplicons showing shifts in the structures between (C), (HS) and (RA) communities and within the density gradient. The different treatments are represented by different colors and symbols as specified in the legend. (D) Bacterial community composition across the density gradient for the three different communities. Relative abundances are shown at the phylum and class levels and expressed as a percentage of the total number of OTUs

The initial gradient in physical distance between cells resulted in differences in α-diversity after 120 days of incubation especially for the control community (C) and the community subjected to ramoplanin (RA) with the lowest diversity indices observed in the smallest microcosms d1 (Fig. 2A and B, Additional file 1: Fig. S1; TukeyHSD test, p-value < 0.05). The impact of physical distancing was weaker for the community exposed to heat-shock (HS) with significant differences observed only for the Shannon and Simpson’s Reciprocal diversity indices. As expected, Principal Coordinates Analysis (PCoA) of the weighted Unifrac distances revealed differences in community structure between the C, HS and RA communities due to the removal treatments (PERMANOVA, *P* < 0.001, R²= 0.69), but also a clear and strong clustering according to the density gradient (PERMANOVA, *P* < 0.001, R²= 0.09) (Fig. 2C and Additional file 1: Table S1). Thus, significant differences were observed between d1 and all the other initial distances for the C, RA and HS communities. We also observed an effect of the interaction between the removal and density treatments (PERMANOVA, *P* < 0.001, R²= 0.05) with significant differences in community structure between d2 and d4 for the C community, but not the RA and HS communities (Additional file 1: Table S2). Clostridia, Gemmatimonadetes and Gamma-protobacteria were mostly affected in the C and RA communities, while the largest changes were observed for the Clostridia, Alpha-proteobacteria and Actinobacteria in the HS community (Fig. 2D). We also quantified the 16S rRNA gene copy numbers using qPCRs as a proxy for bacterial abundance and found the highest number of bacteria at the shortest initial physical distance (d1) in the C community (6.7 × 10^8^ gene copies g^− 1^ dry soil), while no differences were observed between d2, d3 and d4 (3.6 × 10^8^, 3.7 × 10^8^ and 4.3 × 10^8^ gene copies g^− 1^ dry soil, respectively; Additional file 1: Fig. S2). Similarly, the abundances of bacterial communities subjected to heat-shock and ramoplanin were barely affected by physical distancing. These similar numbers of 16S rRNA gene copy per gram of soil, which were in the same range than in the natural soil (4.72 × 10^8^ gene copies g^− 1^ dry soil), also indicate that inoculated communities had completely colonized the microcosms and reached the soil carrying capacity whatever their volumes.

**Fig. 3:**
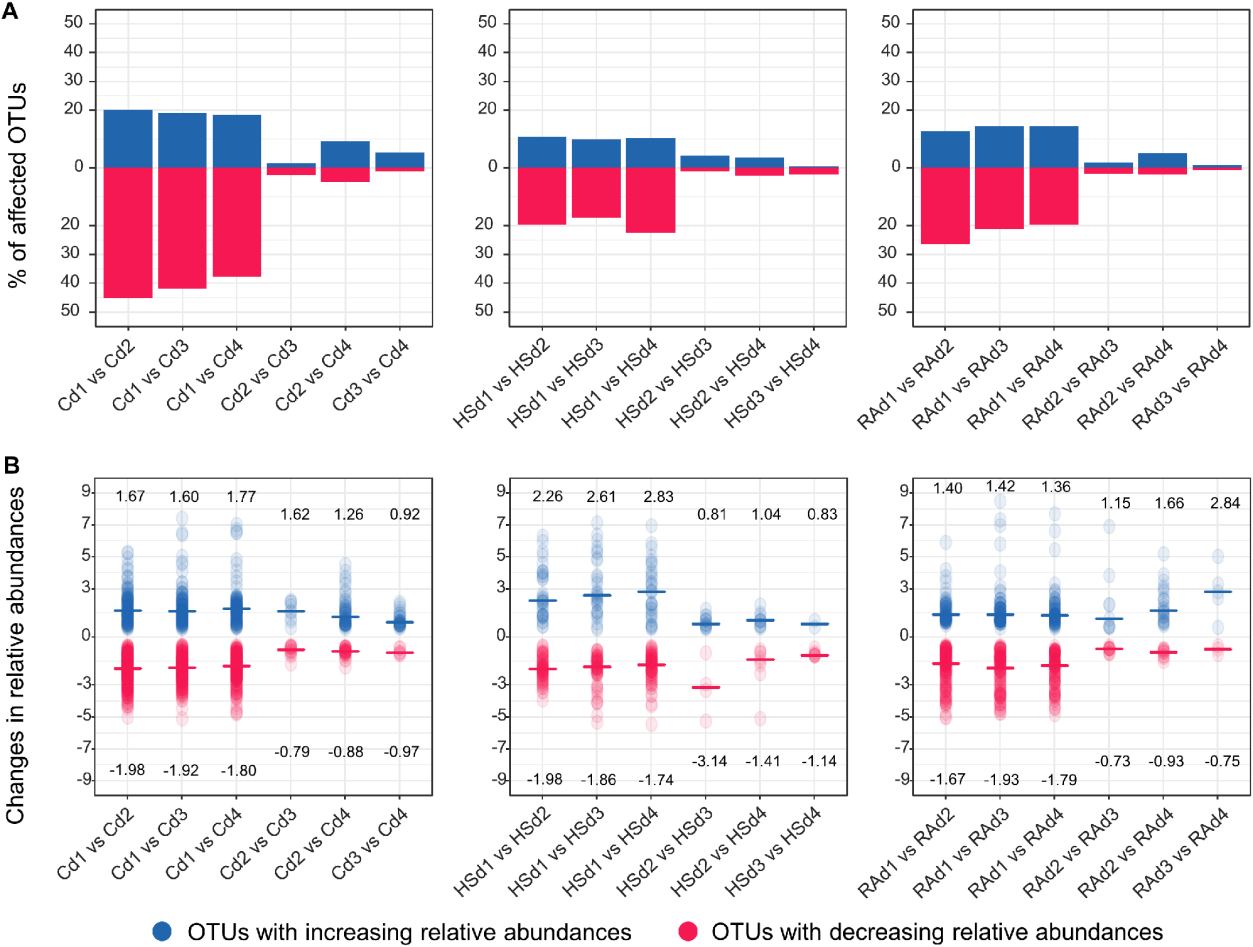
Changes in the relative abundance of the most abundant OTUs related to the physical distancing. Significantly differential abundant OTUs between density treatments (d1, d2, d3 and d4) as identified using a generalized linear mixed model for the control (C), heat-shock (HS) and ramoplanin (RA) communities. (A) Percentage of OTUs exhibiting significantly increasing/decreasing relative abundances for each pairwise comparison between density treatments (where vs. means > or <). (B) Changes in the relative abundances of significantly affected OTUs as represented by the coefficient estimates obtained by the generalized linear mixed model for each OTU within each comparison and used as a measure of the effect size. Median of the coefficient estimates are indicated for each comparison

We expected the shifts in biotic interactions along the gradient in physical distance to be mirrored by changes in OTUs relative abundances, with a higher number of OTUs affected at high initial cell density (i.e. short physical distances). To identify the OTUs affected by our cell physical distancing approach within the C, HS and R communities, we used a generalized linear mixed model estimating significant shifts in the relative abundance of each of the dominant OTUs between density treatments (Fig. 3). Our analysis showed that in total 73%, 41% and 52% of the dominant OTUs were significantly affected by the density treatment for the C, HS and RA communities, respectively (FDR adjusted p-value ≤ 0.05, Additional file 1: Table S3). These differences were mostly observed between the highest cell density (d1) and all other densities. We also found that the number of OTUs with decreasing relative abundance between densities was about two time higher than the number of OTUs with increasing relative abundances, whatever the community (Fig. 3A). OTUs belonged to Gamma-Proteobacteria and Clostridia were mostly positively affected by shorter physical distance, while members of Bacilli, Actinobacteria and Alpha-proteobacteria were negatively affected (Additional file 1: Fig. S3). Overall, the magnitude of the changes in the relative abundances of the significantly affected OTUs was also influenced by the physical distancing and by community memberships. Thus, the magnitude of the changes in relative abundances was stronger for positively impacted OTUs (blue, increasing relative abundances) than for negatively impacted ones (red, decreasing relative abundances) in d1 compared to the other distances for the HS community, while the opposite was found for C and RA communities (Fig. 3B).

### Manipulating cell density leads to modifications in co-occurrence networks

**Fig. 4:**
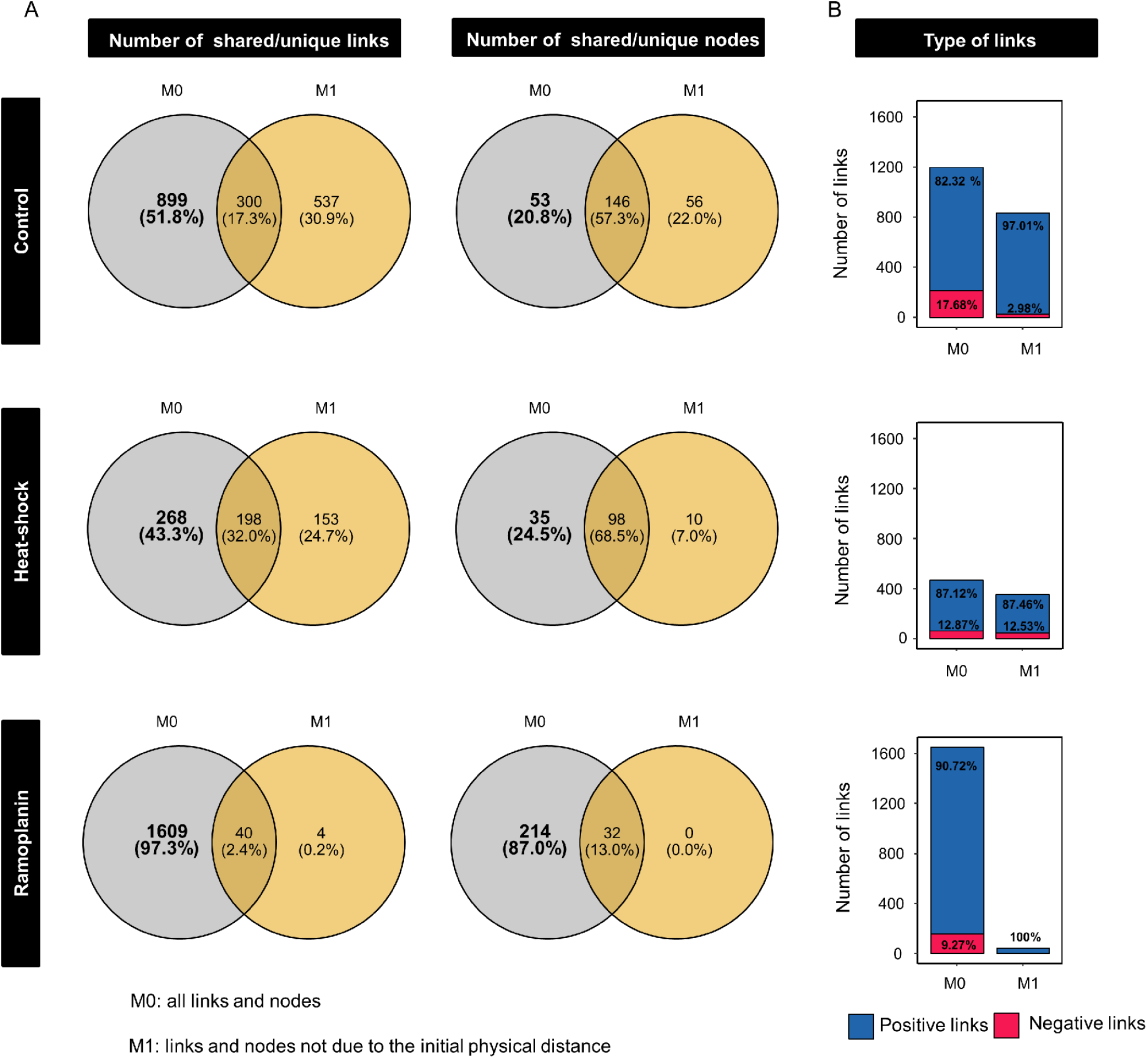
Effects of the physical distancing approach on the microbial co-occurrence networks. (A) The Venn diagrams show the number of shared/unique links or nodes between co-occurrence networks inferred without covariate (M0) or with the initial physical distance as covariate (M1). The nodes and links highlighted in bold correspond to those specifically related to the effect of the physical distancing treatment when comparing the M0 and M1 models. (B) For each network model and community, the number and proportion (out of the total number of links per model network) of positive (blue) and negative (red) links are represented. Links represent partial correlations ρ and they are colored blue if ρ > 0 and red if ρ < 0

To further explore to which extent interactions between bacterial OTUs were influenced by cell density within each community (i.e. control, heat-shock and ramoplanin), we used the initial physical distance as a covariate (M1) for inferring microbial co-occurrence networks, which comprise only associations between OTUs that were not caused by the effects of physical distancing. Comparison of this network to the microbial co-occurrence network built without a covariate (M0 comprising all associations between OTUs) allowed to identify the links between OTUs specifically caused by the effects of physical distancing [49]. We found that 20.8% of nodes and 51.8% of links were specifically related to the effect of the physical distancing for the C community and 24.5% of nodes and 43.3% of links for the HS community. In contrast, 87% of nodes and 97.3% of links were related to the initial physical distance for the RA community (Fig. 4A). Negative links constituted a higher proportion of links that were dependent on physical distancing in the networks inferred from the C (M0: 17.68% vs. M1: 2.98%) and RA communities (M0: 9.27% vs. M1: 0%), compared to the HS community (M0: 12.87% vs. M1: 12.53%) (Fig. 4B). Among the negative links related to the initial physical distance, 90% were connecting Clostridia with either Proteobacteria, Longimicrobia or Bacteroidia in the C network while 72% of negative links in the HS community network were between Clostridia and Bacilli, and 13% between Clostridia and Actinobacteria (Additional file 1: Fig. S4). The depletion of Clostridia in the RA community resulted in shifts in the taxa associations with the highest percentage of negative links (53%) connecting Delta-proteobacteria with mainly Alpha-proteobacteria, Gamma-proteobacteria, Bacilli and Bacteroidia. At the phylum or class taxonomic level, the links between nodes belonging to the same pair of taxa were in some cases both positive and negative (Additional file 1: Fig. S4). However, identifying the nodes corresponding to OTUs exhibiting significant changes in relative abundances allowed to distinguish different families within phylum/classes, resulting in a clearer effect of physical distancing (Additional file 1: Fig. S5). For example, both negative and positive links were observed between the nodes belonging to Clostridia and Bacilli classes in the heat-shock communities (Additional file 1: Fig. S4B), while we noted only negative links between the *Gracilibacteraceae* (Clostridia) and *Paenibacillaceae* (Bacilli) families, as well as only positive links between *Gracilibacteraceae* and *Bacillaceae* (Bacilli) families (Additional file 1: Fig. S5B).

### Coalescence outcomes between communities assembled under different initial physical distances

**Fig. 5:**
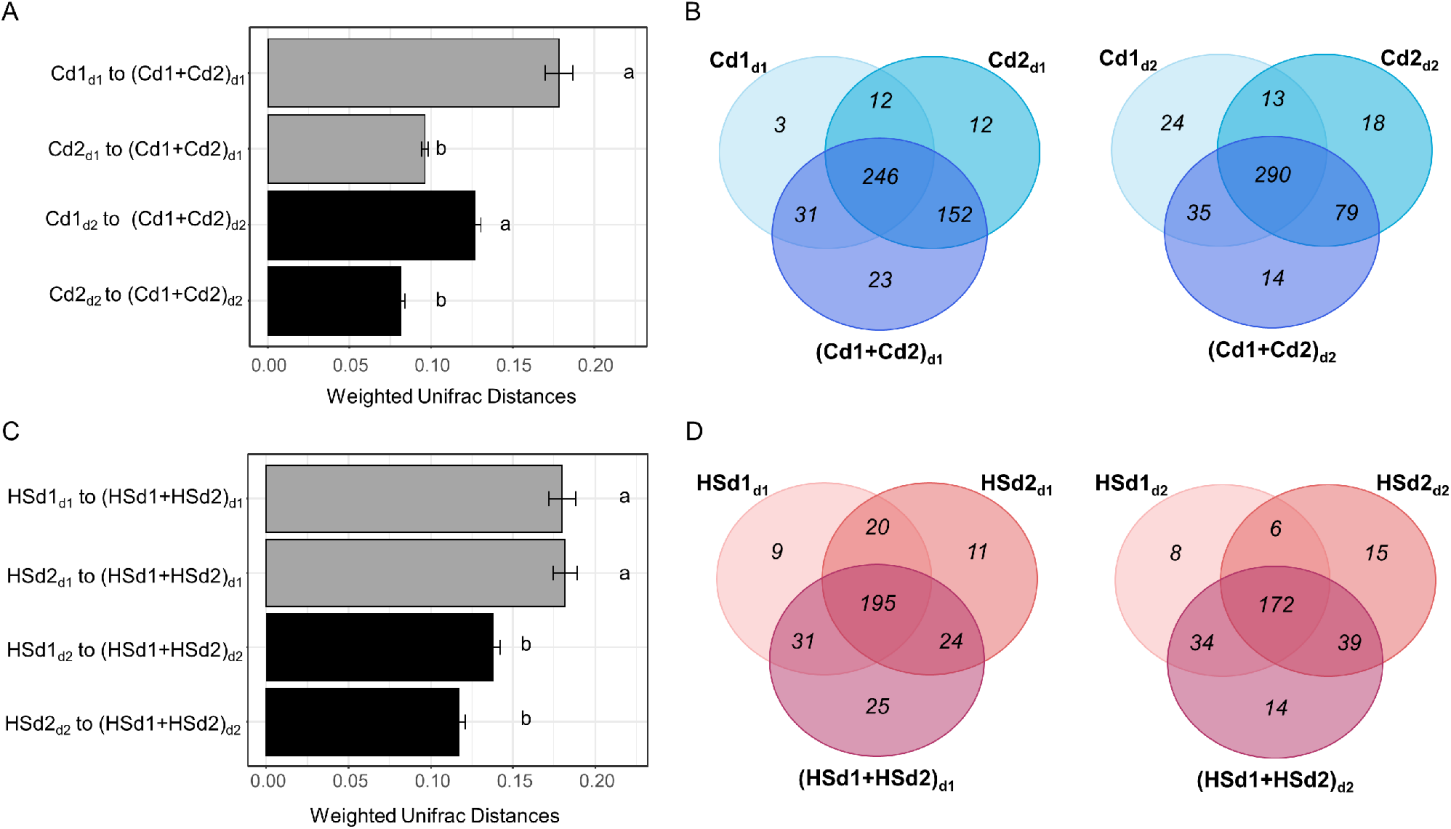
Differences in bacterial community structure and composition between coalesced and reference communities. Weighted UniFrac distances between coalesced and reference communities during the step 2 experiment are represented for the control (A) and heat-shock (C) (mean ± s.e.). Different letters above the bars indicate significant differences according to Nemenyi’s all-pairs comparison test (p-value *<* 0.05). The Venn diagrams show the number of shared/unique OTUs between the coalesced and references communities for the control (B) and heat-shock (D) at high (d1) and low densities (d2)

To determine the extent to which the initial gradient in physical distance selected OTUs with different competitive abilities, we focused on the C and HS communities as well as the d1 and d2 densities for performing a coalescence experiment. The same volumes of soil colonized under the d1 and d2 initial densities were mixed together with two different volumes of sterile soil to again obtain microcosms with short (d1) or long (d2) physical distancing between cells (Fig. 1C). As reference communities, we also used the soils colonized under the d1 and the d2 initial densities but mixed separately with sterile soil at high (d1) or low (d2) densities. After 90 days of soil recolonization, we quantified the outcome of community coalescence by comparing similarities between each reference community and the coalesced community for both densities using weighted Unifrac distances (Additional file 1: Fig. S6 and Table S4). For the C communities, we found that the coalesced communities (Cd1 + Cd2) were more similar to the Cd2 than to the Cd1 reference community whatever the density (Fig. 5A). This coalescence asymmetry was confirmed by a higher proportion of OTUs originating from the Cd2 reference community in the coalesced community at both densities (Fig. 5B). Differential abundance analysis between coalesced and reference communities also showed a higher percentage of impacted OTUs when comparing the coalesced community to the reference community Cd1 (30.90% and 23.43% in d1 and d2, respectively; FDR adjusted p-value ≤ 0.05) than to the reference community Cd2 (13.93% and 12.92% in d1 and d2, respectively; FDR adjusted p-value ≤ 0.05) (Additional file 1: Table S5). In contrast, mixing HS communities that had colonized the soil under the d1 and d2 densities resulted in coalesced communities that were equally similar to the HSd1 and HSd2 reference communities regardless of the density (Fig. 5C). The similar percentage of OTUs shared between the reference and coalesced HS communities also indicated a symmetric coalescence (i.e. none of the source community is predominant in the coalesced community) (Fig. 5D). No effect of the reference community was observed by differential abundance analysis on the outcome of coalescence events between the HS communities (13% and 15.17% of affected OTUs for HSd1 and HSd2, respectively in d1; 6.81% and 7.43% for HSd1 and HSd2, respectively in d2; FDR adjusted p-value ≤ 0.05) (Additional file 1: Table S5). However, we found an effect of the physical distance on the outcome of coalescence events between the HSd1 and HSd2 communities only, with the coalesced community being more similar to the reference communities in d2 than in d1. This importance of physical distancing for the HS community during coalescence was supported by the differential abundance analysis showing that more OTUs were significantly affected at high (average of 14.08% in d1; FDR adjusted p-value ≤ 0.05) than at low densities (7.12% in d2; FDR adjusted p-value ≤ 0.05) whatever the reference community (Additional file 1: Table S5).

Interestingly, the generalized linear mixed model also revealed non additive-effects with a few OTUs exhibiting significantly higher or lower relative abundances in the coalesced communities than in both reference communities (Additional file 1: Fig. S7 and S8). Thus, out of the 343 OTUs exhibiting significantly different relative abundances between the coalesced and reference communities, we found 29 OTUs showing either synergistic or antagonistic non-additive effects in the coalesced community at high cell density (d1), and only 9 OTUs at low cell density (d2).

## Discussion

Although mathematical models have shown that the relative fitness of individuals strongly depends on the density of surrounding neighbors [22, 24], little is known about how biotic interactions are influenced by physical distance between cells in complex environments and their role in microbial community assembly. Here, using removal treatments [8], we generated three microbial inoculums differing in community membership that were then subjected to a physical distancing approach in order to assess to what extent microbial communities are shaped by biotic interactions between neighboring cells. As expected [8], the HS treatment resulted in a decrease in the relative abundances of Gram-negative bacteria, such as γ-Proteobacteria (38% in the control versus 5.5% in the HS treatment). The effect of the RA treatment on microbial communities was less pronounced, yet it led to a decrease in the relative abundances of Gram-positive bacteria, specifically those belonging to Clostridia (4.12% in the control versus 0.6% in the RA treatment). Inoculation of the same microbial pools in microcosms containing different volumes of the same sterilized soil but with the same soil depth and humidity allowed us to control for potential confounding abiotic factors that could interfere with the assessment of the effects of physical distancing. However, our experimental design doesn’t allow controlling for the distribution of the different species, and especially of the rare ones in the community. If this initial species distribution in the microcosms is of importance in determining the outcome of interactions between species and, consequently, bacterial community assembly [43], we should expect a high stochasticity, which increases with physical distancing if species are not evenly distributed. In contrast, we observed a good reproducibility between the 10 replicates whatever the initial distance.

Manipulation of the initial physical distances between bacterial cells successfully highlighted the importance of biotic interactions for bacterial community assembly with at least 41% of the dominant OTUs being affected by cell density. Thus, physical distancing modified the outcome of soil colonization with significant differences both in community diversity and composition that were related to the initial densities. Thus, we found no or minor differences among the initial distances d2, d3 and d4, while the lowest bacterial diversity was observed when the initial physical distance was the shortest (d1). This is in agreement with the classical competition theory, which predicts that environments with higher competition tend to have lower species richness [51]. The higher richness and evenness observed at low initial cell density also provides empirical support for the existence of competition-colonization tradeoffs that could help maintaining bacterial diversity in soil [52]. The identification by differential abundance analysis of a higher percentage of dominant OTUs with decreasing (28%) than increasing relative abundance (14%) at high cell density suggests that reducing the physical distance during soil recolonization increased negative rather than positive interactions. Although cooperation is thought to be a common interaction between species [53], our results support previous studies based on experimental approaches highlighting the importance of competitive interactions in shaping microbial communities [6, 27, 54]. The effect of physical distancing was not linear but threshold based with a stronger effect in d1 versus the other densities and to a lesser extent, in d2 versus d4 (i.e. scenario 3 in the Fig. 1A). This could be explained by the ability of bacteria to detect local cell density through quorum sensing, which can for example repress bacterial competition systems including secretion systems until a threshold density has been reached [31, 55, 56]. It has also been showed that at lower densities, bacteria have more opportunities during range expansion to form established clonal patches, which are more protected from competitors [57, 58]. Inferring microbial networks with and without the initial density gradient as covariate allowed us to identify the microbial associations that were directly related to the effect of physical distancing. In any case, since we used the same soil, which was incubated under the same conditions, abiotic filtering was intrinsically limited by our approach. Overall, a higher proportion of negative links explained by the initial density gradient in the inferred microbial co-occurrence networks further supports that competitive interactions were more affected by physical distancing for C and RA communities compared to the HS community. Among the co-occurrence networks, we found that Clostridia were often negatively associated with Proteobacteria and Bacilli and that these associations were specifically related to physical distancing. This is consistent with previous findings reporting that, in soil, members of Clostridia could produce antimicrobial compounds which negatively affected the growth of species belonging to *Pseudomonas* and *Bacillus* [59]. However, future studies are required for understanding the competition mechanisms underpinning the microbial interactions highlighted in our work.

The generation of three different microbial communities using removal treatments allowed us to characterize to which extent the effect of physical distancing was dependent on community composition. Specifically, we found a higher percentage of OTUs with decreasing fitness in the C- and RA-communities compared to the HS community, which was concomitant with a higher number of negative links related to the physical distancing in C and RA bacterial networks. Inferring networks with or without the initial physical distance as a qualitative covariate also revealed that RA community network was the most responsive to the initial neighboring cell density. This could be due to the enrichment of Gram-negative bacteria (Fig. 2d) after exposure to ramoplanin, an antibiotic with bactericidal activity against Gram-positive bacteria. Accordingly, many secretion systems involving cell contact or cell-cell communication through quorum sensing and playing a pivotal role in bacterial competition have been described only in Gram-negative bacteria [60]. Taken together, our results suggests that physical distancing could differentially modulate competitiveness between surrounding species depending on community membership.

To further explore how physical distancing affects interactions within microbial communities, we used a coalescence experiment based on the mixing of communities that assembled at high (d1) and low (d2) cell densities. We found that coalescence events resulted in distinct patterns for C and HS communities with the source community being more important for the assembly of the C-coalesced communities while the physical distance was more important for that of the HS-coalesced communities. While we hypothesized that increasing physical distance will favor poor competitors, the Cd2 source community was dominating over the Cd1 source community within the C-coalesced communities assembled at both high and low densities. This scenario can be explained with the findings of Lechón-Alonso et al. [61] who showed that the less competitive parent communities can dominate after coalescence when they are more cooperative because of their superior ability to deplete resources. Conversely, we found a symmetric coalescence for the HS communities indicating that the HSd1 and HSd2 communities were equally competitive, which suggests that physical distancing during step1 experiment had little effect on their competitiveness. This is supported by the much weaker effect of physical distancing on the HS than on C communities during the step1 experiment with about 36% and 69% of significantly affected OTUs between d1 and d2, respectively. This lack of “home field advantage” during coalescence with the communities selected at the d2 density being equally or more competitive than the d1 community even when mixed at the d1 density suggests that the observed differences in competitiveness were due to biotic rather than abiotic filtering during the first step of physical distancing approach.

Using the coalescence approach, we also identified OTUs with significantly higher or lower relative abundances in the coalesced communities compared to the reference communities. These antagonistic and synergistic effects resulting from the mixing of partly different communities could be due to shifts in the initial abundance of the interacting cells in the coalesced communities [62]. Alternatively, the introduction during the coalescence of new species present only in one of the parent communities may have modified the existing interaction in the other parent community. Accordingly, the importance of such higher-order interactions is increasingly recognized in microbial community assembly [63]. Interestingly, we found that these antagonistic and synergistic interactions also occur more often under short than long physical distancing, which further supports the importance of neighboring cell density for biotic interaction frequency.

## Conclusions

In summary, by experimentally manipulating the physical distance between neighboring cells, our study showed the importance of biotic interactions in microbial community assembly. Reducing the initial distances between cells led to a loss of bacterial diversity, with at a higher percentage of OTUs exhibiting a decrease than an increase in relative abundance, therefore suggesting a predominance of negative interactions. However, the differential effect of physical distancing observed between the generated inoculums suggests that community membership either modulates the importance of biotic interactions in community assembly or the extent to which biotic interactions are dependent on neighboring cell density. Further studies are therefore needed to resolve microbe-microbe interactions within complex communities, which is crucial for steering microbial communities in the environment.

### Electronic supplementary material

Below is the link to the electronic supplementary material.

**Supplementary Material 1: Additional file 1. Table S1.** Analysis of differences between treatments based on the weighted Unifrac distances. PERMANOVA results assessing differences in the bacterial community structure linked to removal treatments, density treatments and their interactions using weighted UniFrac distance of step1 experiment. **Table S2.** Analysis of differences between density treatments based on the weighted Unifrac distances. Pairwise comparisons assessing differences in the bacterial community structure related to the density treatment in the control (C), heat-shock (HS) and ramoplanin (RA) communities using the weighted UniFrac distances with Benjamini–Hochberg corrections for multiple testing. **Table S3.** Identification of significantly affected OTUs for step1 experiment. Results from differential abundance analysis of OTUs within each community using a generalized linear mixed model (FDR adjusted p-value ≤ 0.05). **Table S4.** Analysis of differences between treatments based on the weighted Unifrac distances. PERMANOVA results assessing differences in the bacterial community structure linked to the community, density and their interactions using weighted UniFrac distance of step2 experiment. **Table S5.** Identification of significantly affected OTUs for step2 experiment. Results of differential abundance analysis of OTUs between coalesced and references communities using a generalized linear mixed model (FDR adjusted p-value ≤ 0.05). **Fig. S1** Diversity levels of the bacterial community after step 1 experiment. The Faith’s phylogenetic diversity (A) and Shannon (B) indices are shown (mean ± s.e.) in the control (C), heat-shock (HS) and ramoplanin (RA) communities within the density gradient (d1, d2, d3 and d4). Different letters indicate significant differences according to TukeyHSD test (p-value < 0.05). **Fig. S2** Quantification of the total bacterial community. Abundances of total bacteria (16 S rRNA) in the control (C), heat-shock (HS) and ramoplanin (RA) communities within the density gradient (d1, d2, d3 and d4) after Step 1 experiment (mean ± s.e. of log10-transformed data expressed as gene copy g^-1^ dry soil). Different letters above the bars indicate significant differences according to TukeyHSD test (p-value < 0.05). **Fig. S3** Phylogenetic relationships and distribution of significantly affected OTUs by the physical distancing approach. Significantly increasing/decreasing relative abundances of OTUs between density treatments according to the generalized linear mixed model for the control, heat-shock and ramoplanin communities. Changes in the relative abundances as measured by the coefficient estimates (effect size) are represented by the blue-to-red color. The affiliation of OTUs at the phylum or class levels is indicated by different colors on the internal ring. **Fig. S4** Effects of the physical distancing approach on the microbial co-occurrence networks. Number of positive (blue) and negative (red) links that are related to the physical distance (M0-M1) for the control (A), heat-shock (B) and ramoplanin (C) communities. The Venn Diagrams show the number of links that are related to the physical distance (M0-M1). For visualization purpose, only taxa with number of links higher than the average number of neighbors in each community network (M0) was represented. **Fig. S5** Nodes related to physical distancing in co-occurrence networks and significantly affected by the physical distancing approach. Number of positive (blue) and negative (red) links between nodes that are related to physical distance in co-occurrence networks (M0-M1) and exhibiting significant changes in relative abundances as determined by the differential abundance analysis for the control (A), heat-shock (B) and ramoplanin (C) communities. **Fig. S6** Differences in bacterial community composition across treatments for the step2 experiment. Principal Coordinates Analysis (PCoA) of the weighted UniFrac distance matrix of 16 S rRNA gene amplicons of coalesced and references communities for the control (A) and heat-shock (B) at high (d1) and low densities (d2). The different treatments are represented by different colors and symbols as specified in the legend. **Fig. S7** Identification of OTUs with significantly lower or higher relative abundances in the coalesced communities compared to the reference communities for the control. OTUs exhibiting significant differences in the coalesced communities compared to the reference communities as identified by the generalized linear mixed model at high (d1) and low densities (d2). Relative abundances are shown at the family level and the affiliation of OTUs are indicated by different colors at the phylum or class levels. **Fig. S8** Identification of OTUs with significantly lower or higher relative abundances in the coalesced communities compared to the reference communities for the heat shock. OTUs exhibiting significant differences in the coalesced communities compared to the reference communities as identified by the generalized linear mixed model at high (d1) and low densities (d2). Relative abundances are shown at the family level and the affiliation of OTUs are indicated by different colors at the phylum or class levels.

## Data Availability

Raw sequences were deposited at the NCBI under the accession number BioProject PRJNA883551. All data are available in the main text or the supplementary information.
