# Supplementary material for: Manipulating the physical distance between cells during soil colonization reveals the importance of biotic interactions in microbial community assembly
Source: Environ Microbiome. 2024 Mar 19;19:18. doi: 10.1186/s40793-024-00559-4 (PMC10953230; doi:10.1186/s40793-024-00559-4)
Supplement: Supplementary file 1 — Supplementary Material 1: Additional file 1. Table S1. Analysis of differences between treatments based on the weighted Unifrac distances. PERMANOVA results assessing differences in the bacterial community structure linked to removal treatments, density treatments and their interactions using weighted UniFrac distance of step1 experiment. Table S2. Analysis of differences between density treatments based on the weighted Unifrac distances. Pairwise comparisons assessing differences in the bacterial community structure related to the density treatment in the control (C), heat-shock (HS) and ramoplanin (RA) communities using the weighted UniFrac distances with Benjamini–Hochberg corrections for multiple testing. Table S3. Identification of significantly affected OTUs for step1 experiment. Results from differential abundance analysis of OTUs within each community using a generalized linear mixed model (FDR adjusted p-value ≤ 0.05). Table S4. Analysis of differences between treatments based on the weighted Unifrac distances. PERMANOVA results assessing differences in the bacterial community structure linked to the community, density and their interactions using weighted UniFrac distance of step2 experiment. Table S5. Identification of significantly affected OTUs for step2 experiment. Results of differential abundance analysis of OTUs between coalesced and references communities using a generalized linear mixed model (FDR adjusted p-value ≤ 0.05). Fig. S1 Diversity levels of the bacterial community after step 1 experiment. The Faith’s phylogenetic diversity (A) and Shannon (B) indices are shown (mean ± s.e.) in the control (C), heat-shock (HS) and ramoplanin (RA) communities within the density gradient (d1, d2, d3 and d4). Different letters indicate significant differences according to TukeyHSD test (p-value < 0.05). Fig. S2 Quantification of the total bacterial community. Abundances of total bacteria (16 S rRNA) in the control (C), heat-shock (HS) and ramoplanin (RA) com [file 40793_2024_559_MOESM1_ESM.pdf]

Supplementary material

Additional file 1:

Manipulating the physical distance between cells reveals the importance of biotic interactions in soil microbial community assembly

Sana Romdhane[1*], Sarah Huet[1], Aymé Spor[1], David Bru[1], Marie-Christine Breuil[1] and Laurent Philippot[1]

[1] Univ. Bourgogne Franche-Comté, INRAE, Institut Agro, Agroécologie, F-21000 Dijon, France

Email Addresses for all authors:

Sana Romdhane: sana.romdhane@inrae.fr

Sarah Huet: sarah.huet@inrae.fr

Ayme Spor: ayme.spor@inrae.fr

David Bru: david.bru@inrae.fr

Marie-Christine Breuil: marie-christine.breuil@inrae.fr

Laurent Philippot: laurent.philippot@inrae.fr

*Corresponding author: sana.romdhane@inrae.fr

Table S1. Analysis of differences between treatments based on the weighted Unifrac distances. PERMANOVA results assessing differences in the bacterial community structure linked to removal treatments, density treatments and their interactions using weighted UniFrac distance of step1 experiment.

	Df	***Sums Of Sqs***	***MeanSqs***	***F.Model***	***R2***	***P value***
Removal Treatment	2	3.5275	1.76376	243.089	0.6961	**< 0.001**
Density Treatment	3	0.4824	0.16078	22.160	0.09512	**< 0.001**
Removal x Density	6	0.2776	0.04627	6.377	0.05474	**< 0.001**
Residuals	108	0.7836	0.00726	-	0.15452	-
Total	119	5.0711	-	-	1.00000	-

Significant terms are in bold

Table S2. Analysis of differences between density treatments based on the weighted Unifrac distances. Pairwise comparisons assessing differences in the bacterial community structure related to the density treatment in the control (C), heat-shock (HS) and ramoplanin (RA) communities using the weighted UniFrac distances with Benjamini–Hochberg corrections for multiple testing.

	Sums Of Sqs	*F.Model*	*R2*	*P adujsted*
**Control**				
d1 vs d2	0.906	111.27871	0.86076594	**0.002**
d1 vs d3	0.842	94.1304424	0.83947267	**0.002**
d1 vs d4	0.751	75.9496674	0.80840805	**0.002**
d2 vs d3	0.007	1.26845068	0.06583044	0.281
d2 vs d4	0.027	3.97500725	0.18088764	**0.015**
d3 vs d4	0.010	1.3346381	0.06902835	0.281
**Heat shock**				
d1 vs d2	0.328	80.5692796	0.81738732	**0.002**
d1 vs d3	0.169	6.80473156	0.274332	**0.002**
d1 vs d4	0.290	49.0635058	0.73159769	**0.002**
d2 vs d3	0.082	3.27800894	0.15405619	0.087
d2 vs d4	0.015	2.57913893	0.12532784	0.087
d3 vs d4	0.095	3.57390541	0.16565871	0.087
**Ramoplanin**				
d1 vs d2	0.492	38.7171571	0.68263572	**0.002**
d1 vs d3	0.486	32.734357	0.64521084	**0.002**
d1 vs d4	0.403	29.5890591	0.6217618	**0.002**
d2 vs d3	0.006	0.94818479	0.05004093	0.493
d2 vs d4	0.012	2.18703831	0.10833874	0.121
d3 vs d4	0.005	0.68869231	0.03685075	0.540

Significant terms are in bold

Table S3. Identification of significantly affected OTUs for step1 experiment. Results of differential abundance analysis of OTUs within each community using a generalized linear mixed model (FDR adjusted p-value ≤ 0.05).

	Number of most abundant OTUs	***Number of significantly affected OTUs***	***% of significantly affected OTUs***
**Control**	529	389	73.5
**Heat-shock**	306	126	41.17
**Ramoplanin**	468	245	52.35

Table S4. Analysis of differences between treatments based on the weighted Unifrac distances. PERMANOVA results assessing differences in the bacterial community structure linked to the community, density and their interactions using weighted UniFrac distance of step2 experiment.

	Df	***Sums Of Sqs***	***F.Model***	***R2***	***P value***
**Control**					
Community	2	0.16120507	18.713436	0.40648813	**< 0.001**
Density Treatment	1	0.06480913	15.0808382	0.16379092	**< 0.001**
Community x Density	2	0.06625069	7.78300166	0.16906023	**< 0.001**
Residuals	24	0.10131031	-	0.26066072	-
Total	29	0.4035752	-	1	-
**Heat-shock**					
Community	2	0.09315436	6.00074563	0.24976919	**< 0.001**
Density Treatment	1	0.05853254	7.54100776	0.15693978	**< 0.001**
Community x Density	2	0.04275121	2.7539141	0.11462624	**0.002**
Residuals	23	0.17852367	-	0.47866479	-
Total	28	0.37296178	-	1	-

Significant terms are in bold

Table S5. Identification of significantly affected OTUs for step2 experiment. Results of differential abundance analysis of OTUs between coalesced and references communities using a generalized linear mixed model (FDR adjusted p-value ≤ 0.05).

	Number of significant OTUs	***% of significant OTUs***
**Control**		
$(Cd1+Cd2)_{d1}$ vs $Cd1_{d1}$	153	30.90
$(Cd1+Cd2)_{d1}$ vs $Cd2_{d1}$	69	13.93
$(Cd1+Cd2)_{d2}$ vs $Cd1_{d2}$	116	23.43
$(Cd1+Cd2)_{d2}$ vs $Cd2_{d2}$	64	12.92
**Heat-shock**		
$(HSd1+HSd2)_{d1}$ vs $HSd1_{d1}$	42	13.00
$(HSd1+HSd2)_{d1}$ vs $HSd2_{d1}$	49	15.17
$(HSd1+HSd2)_{d2}$ vs $HSd1_{d2}$	22	6.81
$(HSd1+HSd2)_{d2}$ vs $HSd2_{d2}$	24	7.43

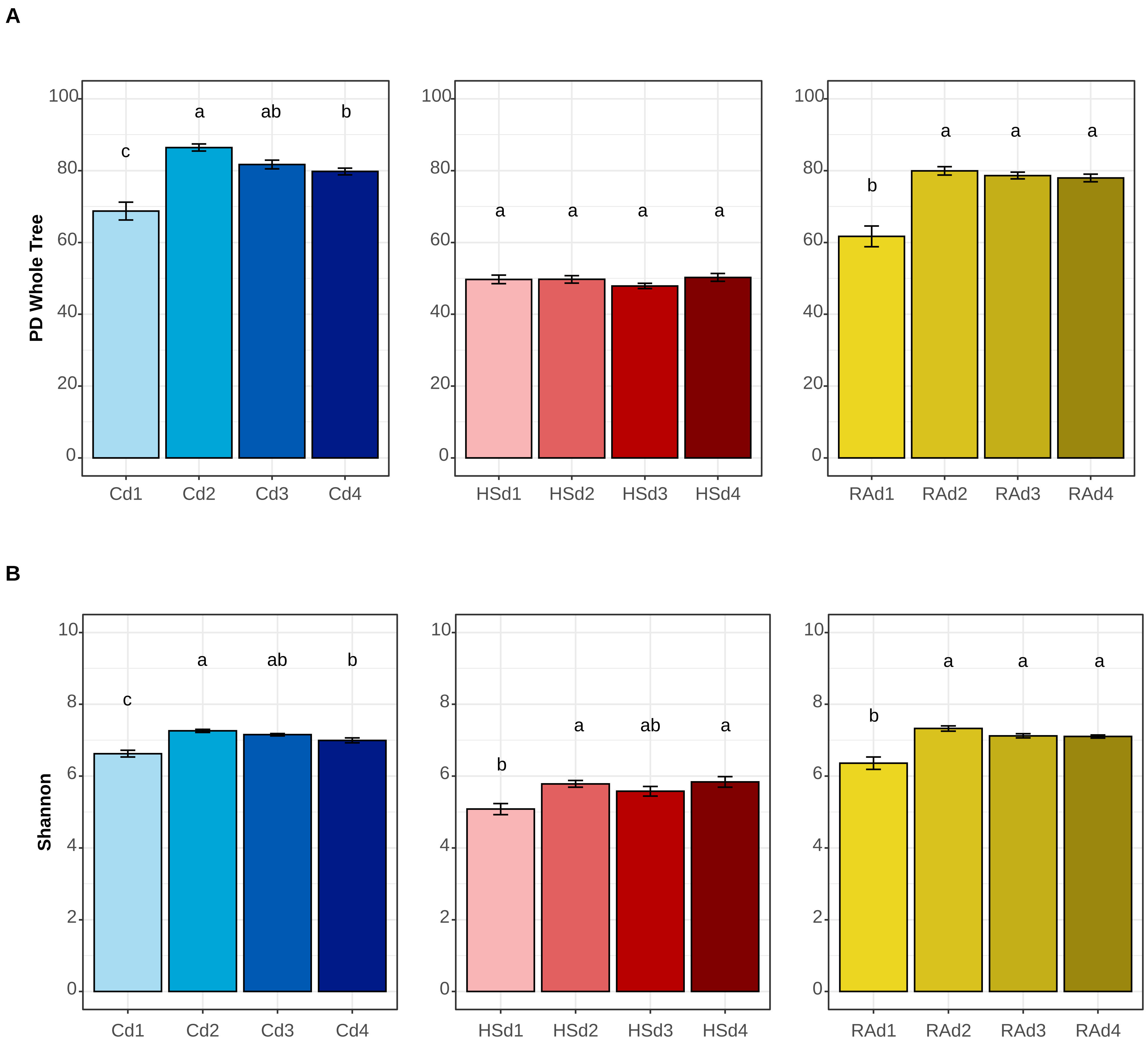

Fig. S1 Diversity levels of the bacterial community after step 1 experiment. The Faith's phylogenetic diversity (A) and Shannon (B) indices are shown (mean ± s.e.) in the control (C), heat-shock (HS) and ramoplanin (RA) communities within the density gradient (d1, d2, d3 and d4). Different letters indicate significant differences according to TukeyHSD test (p-value < 0.05)

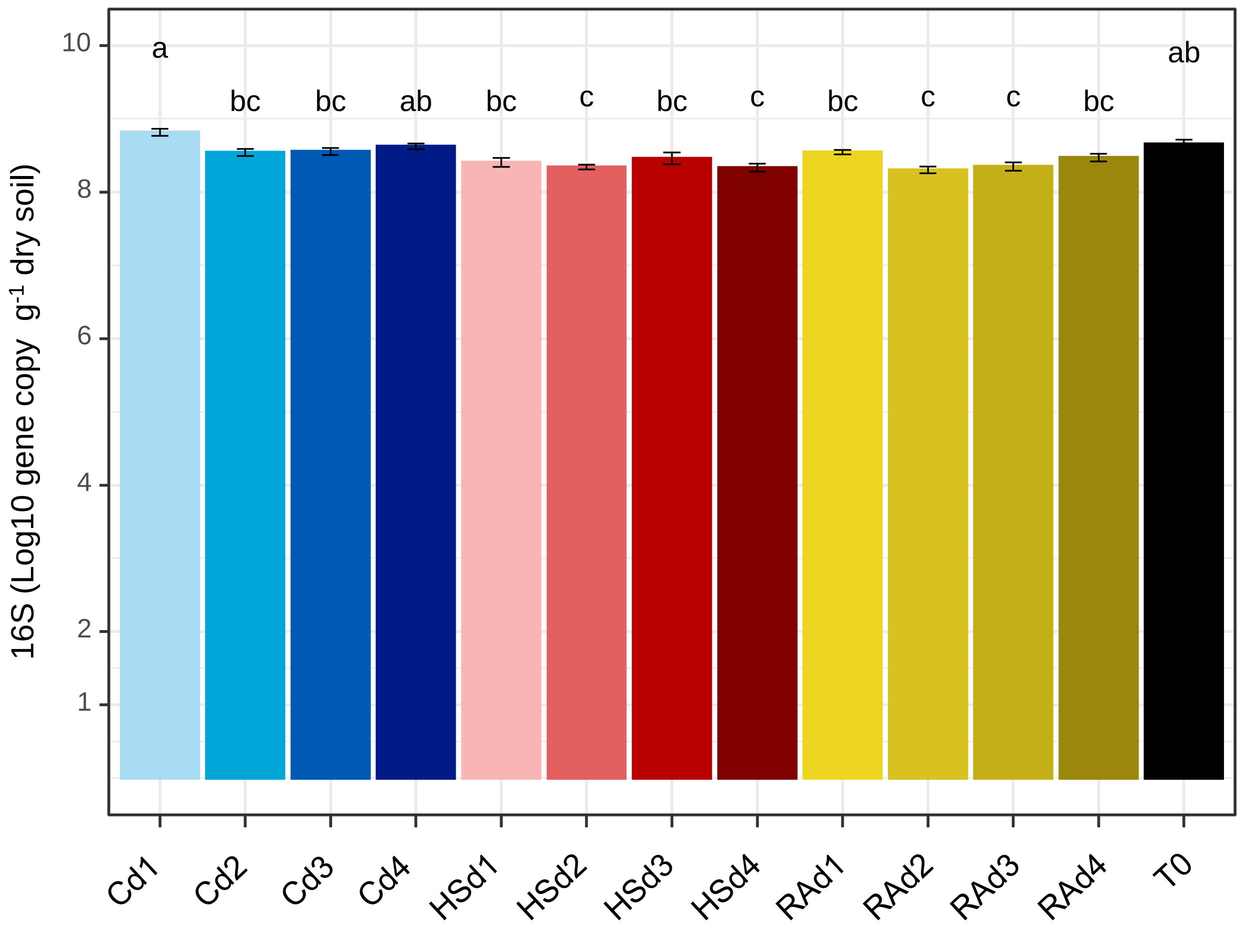

Fig. S2 Quantification of the total bacterial community. Abundances of total bacteria (16S rRNA) in the control (C), heat-shock (HS) and ramoplanin (RA) communities within the density gradient (d1, d2, d3 and d4) after Step 1 experiment (mean ± s.e. of log10-transformed data expressed as gene copy g^{-1} dry soil). Different letters above the bars indicate significant differences according to Tukey's test (p-value < 0.05).

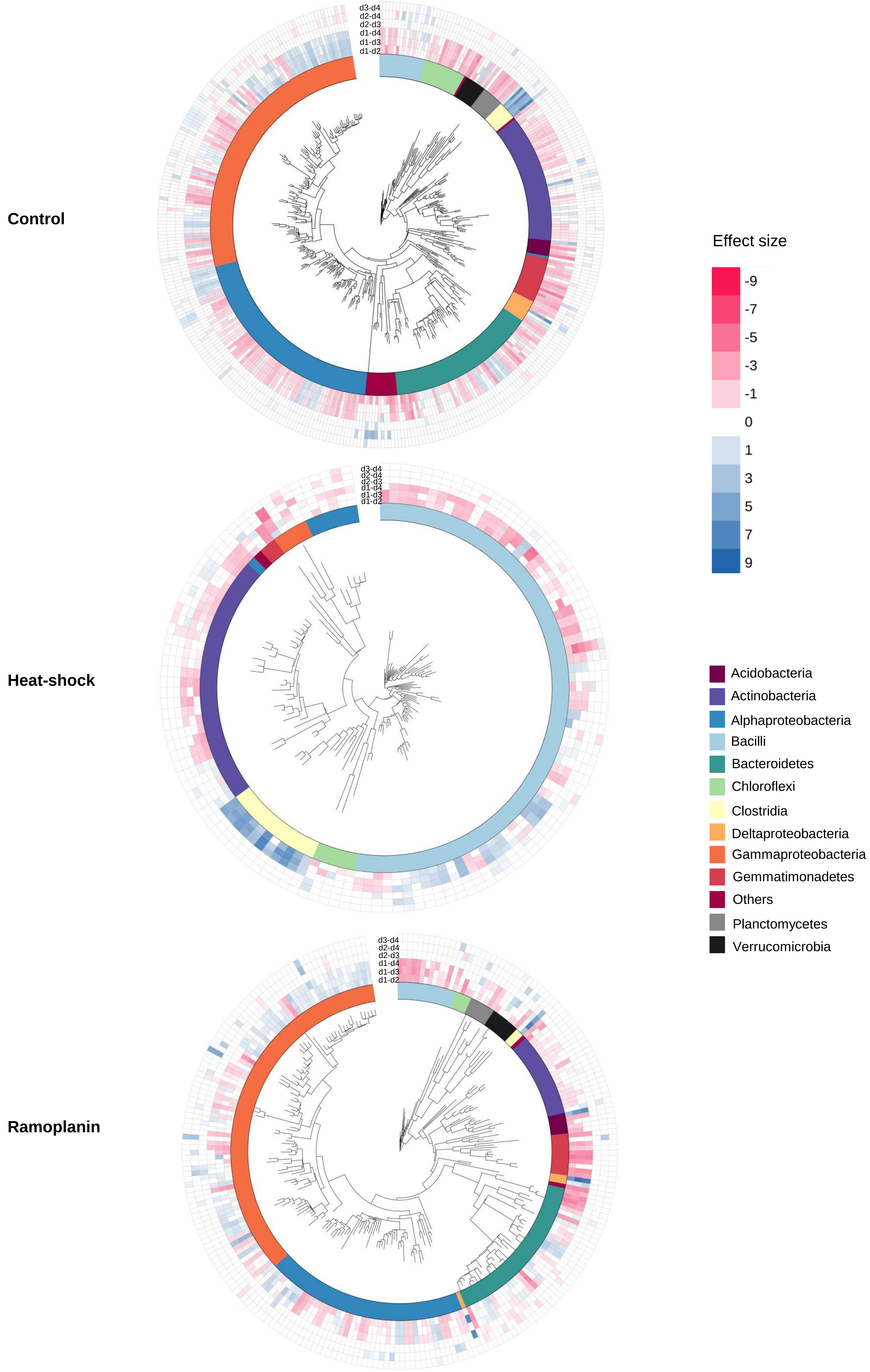

Fig. S3 Phylogenetic relationships and distribution of significantly affected OTUs by the physical distancing approach. Significantly increasing/decreasing relative abundances of OTUs between density treatments according to the generalized linear mixed model for the control, heat-shock and ramoplanin communities. Changes in the relative abundances as measured by the coefficient estimates (effect size) are represented by the blue-to-red color. The affiliation of OTUs at the phylum or class levels is indicated by different colors on the internal ring.

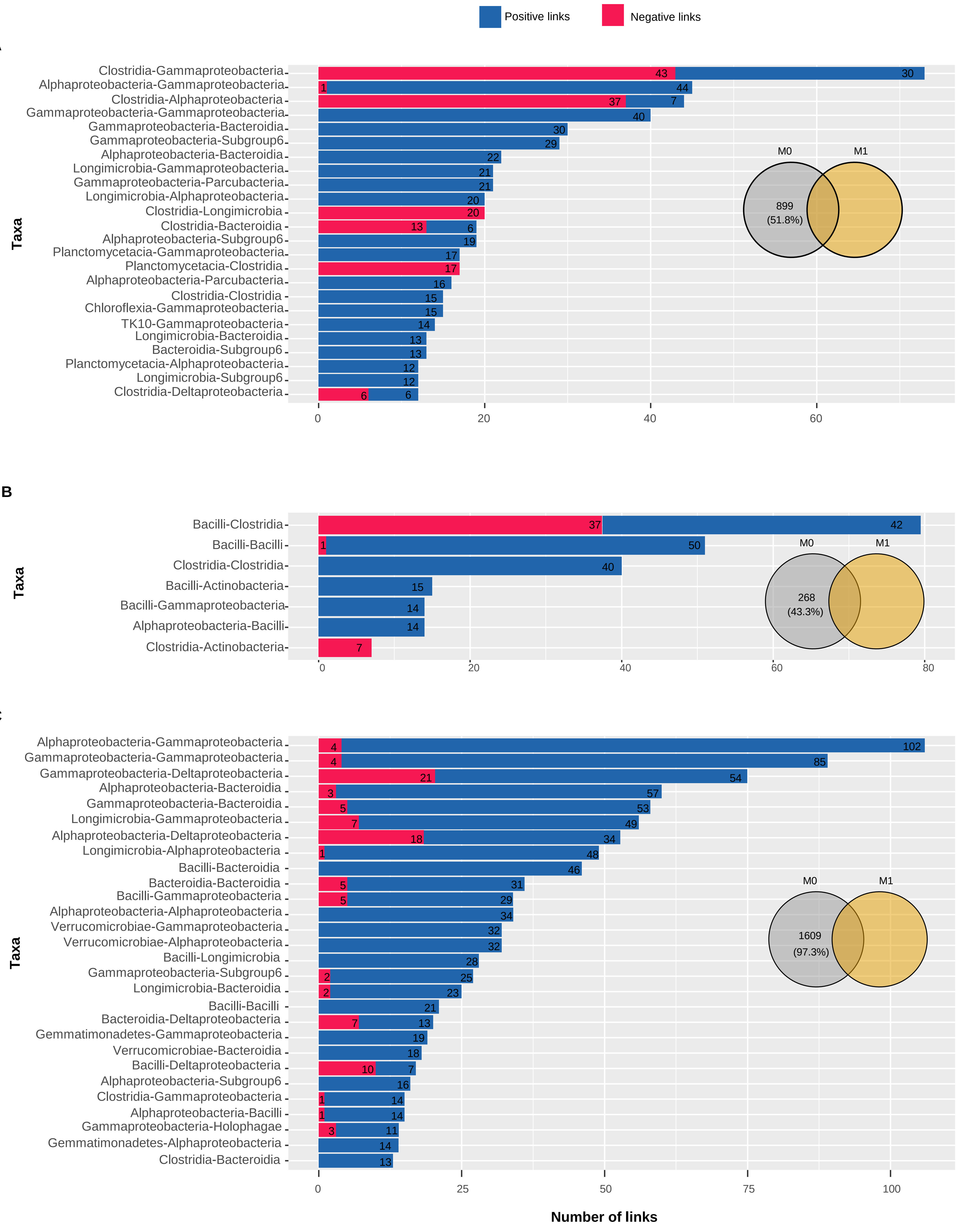

Fig. S4 Effects of the physical distancing approach on the microbial co-occurrence networks. Number of positive (blue) and negative (red) links that are related to the physical distance (M0-M1) for the control (A), heat-shock (B) and ramoplanin (C) communities. The Venn Diagrams show the number of links that are related to the physical distance (M0-M1). For visualization purpose, only taxa with number of links higher than the average number of neighbors in each community network (M0) was represented.

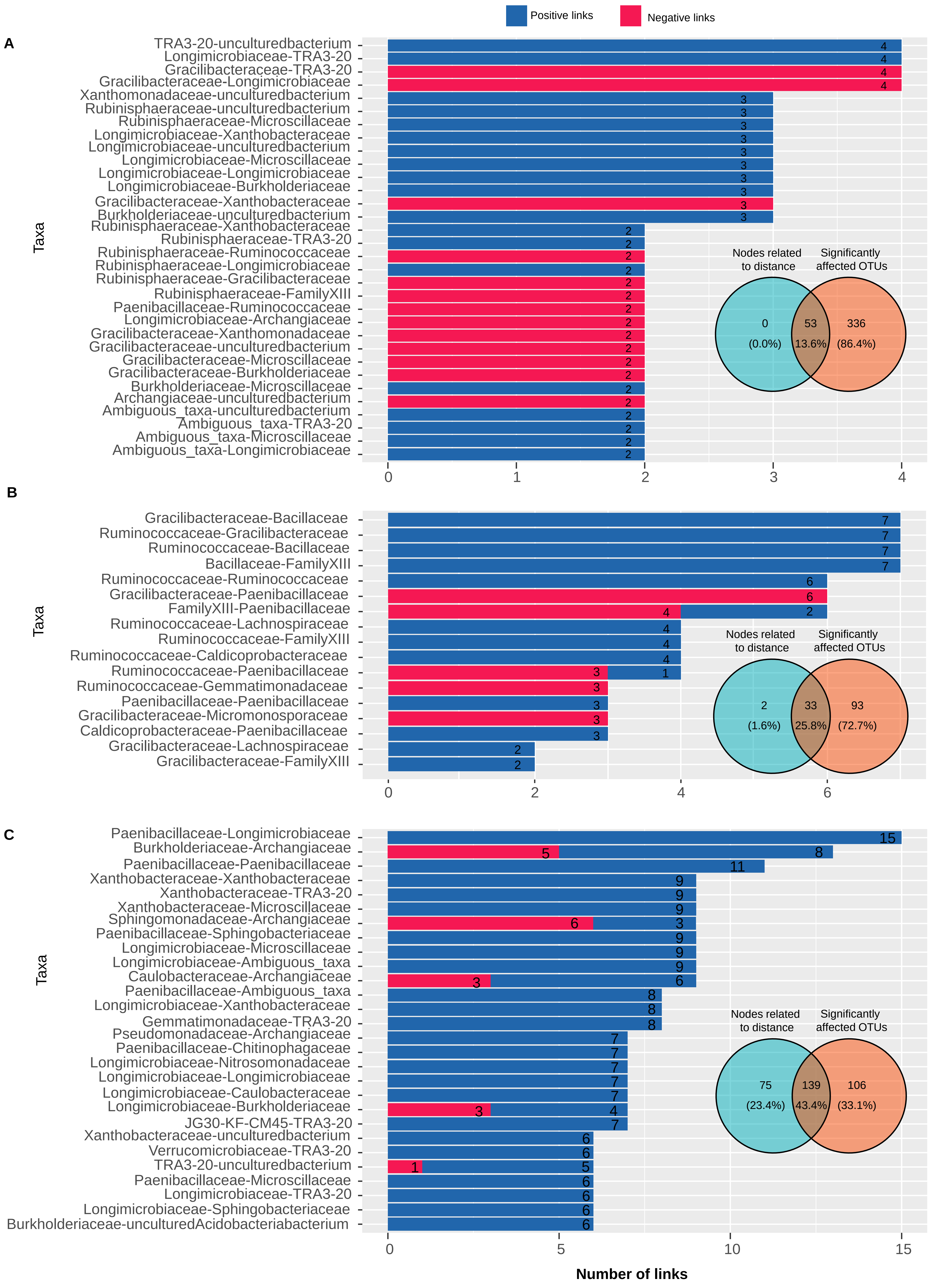

Fig. S5 Nodes related to physical distancing in co-occurrence networks and significantly affected by the physical distancing approach. Number of positive (blue) and negative (red) links between nodes that are related to physical distance in co-occurrence networks (M0-M1) and exhibiting significant changes in relative abundances as determined by the differential abundance analysis for the control (A), heat-shock (B) and ramoplanin (C) communities.

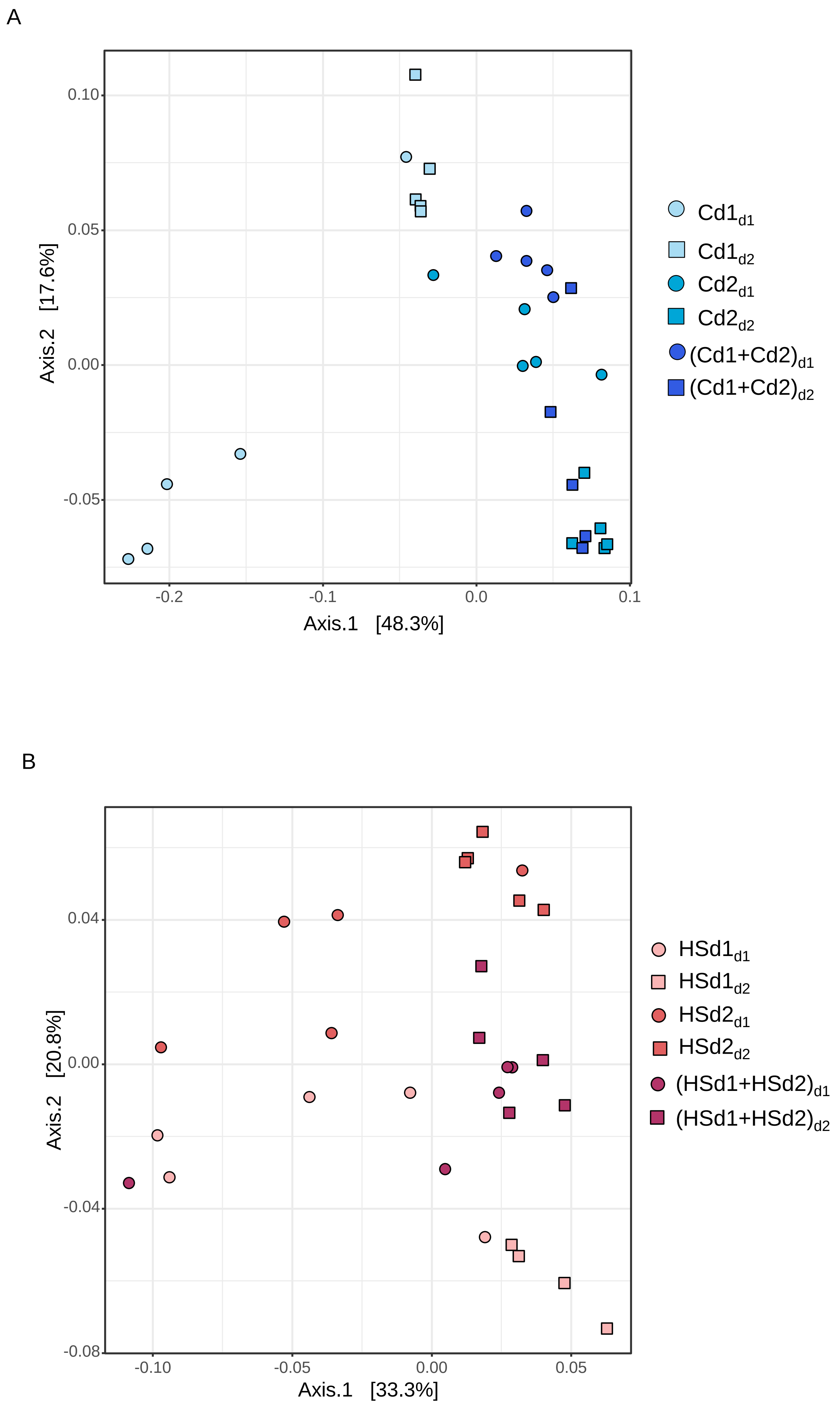

Fig. S6 Differences in bacterial community composition across treatments for the step2 experiment. Principal Coordinates Analysis (PCoA) of the weighted UniFrac distance matrix of 16S rRNA gene amplicons of coalesced and references communities for the control (A) and heat-shock (B) at high (d1) and low densities (d2). The different treatments are represented by different colors and symbols as specified in the legend.

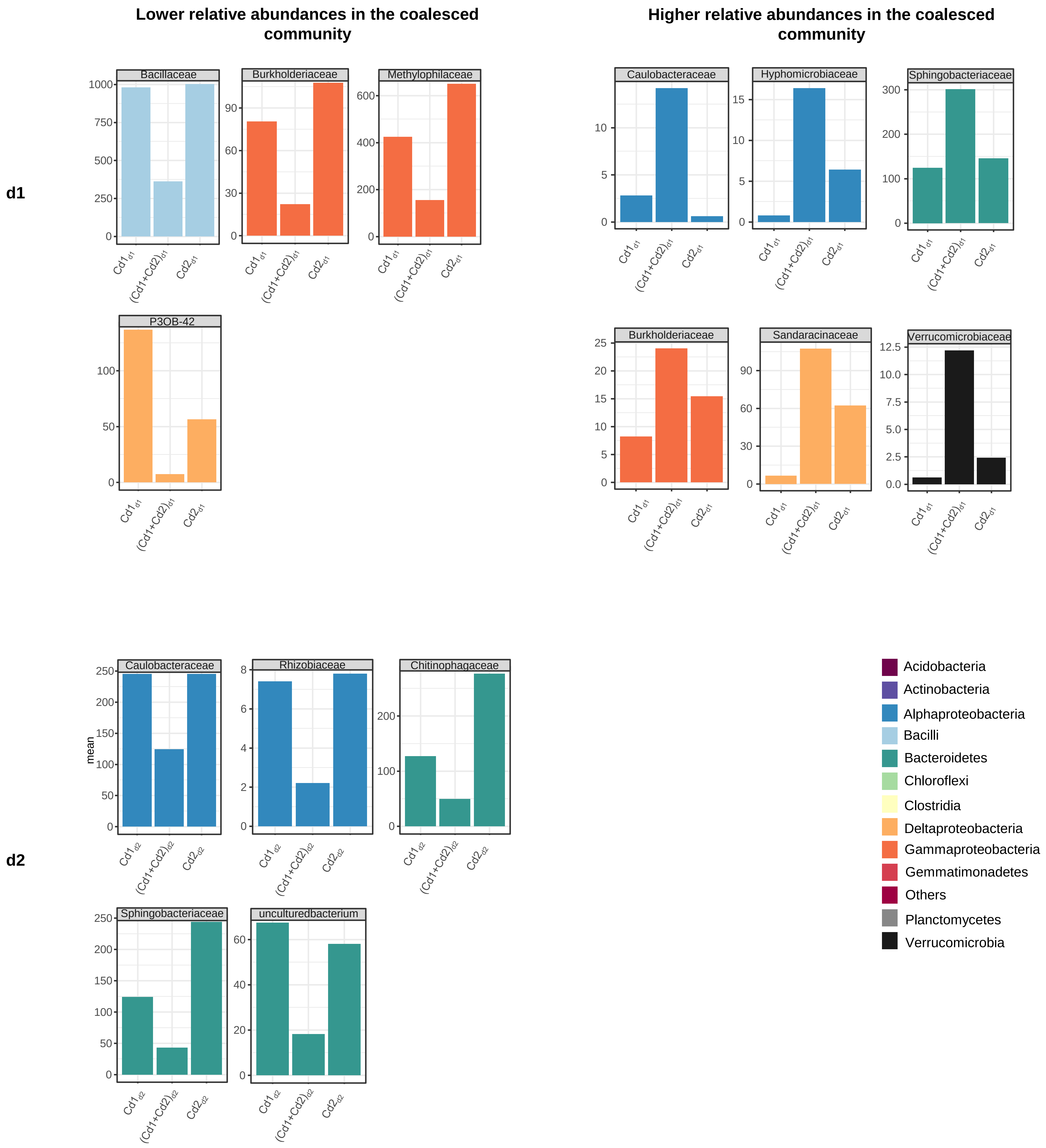

Fig. S7 Identification of OTUs with significantly lower or higher relative abundances in the coalesced communities compared to the reference communities for the control. OTUs exhibiting significant differences in the coalesced communities compared to the reference communities as identified by the generalized linear mixed model at high (d1) and low densities (d2). Relative abundances are shown at the family level and the affiliation of OTUs are indicated by different colors at the phylum or class levels.

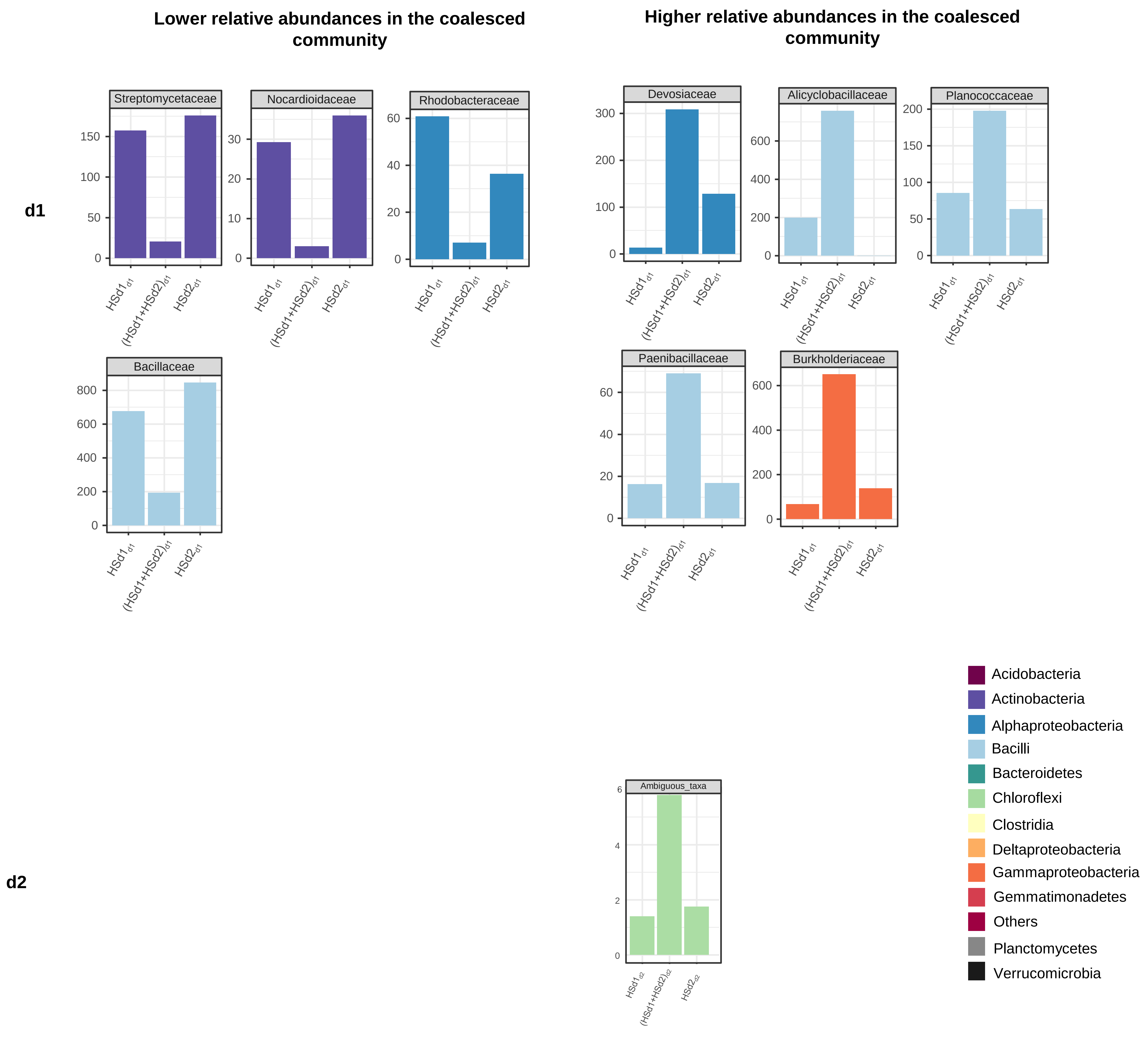

Fig. S8 Identification of OTUs with significantly lower or higher relative abundances in the coalesced communities compared to the reference communities for the heat shock. OTUs exhibiting significant differences in the coalesced communities compared to the reference communities as identified by the generalized linear mixed model at high (d1) and low densities (d2). Relative abundances are shown at the family level and the affiliation of OTUs are indicated by different colors at the phylum or class levels.
